# Intravascular Imaging-Guided Percutaneous Coronary Intervention for Calcified Coronary Lesions: A Network Meta-Analysis

**DOI:** 10.1016/j.jacadv.2026.103237

**Published:** 2026-09-17

**Authors:** Giuseppe Panuccio, Youssef S. Abdelwahed, Giuseppe Tartaglione, Nicole Carabetta, Martina Sportelli, Mario Giovanni Genovese, Jolanda Sabatino, Elaaha Anwari, Kambis Mashayekhi, Emmanouil S. Brilakis, Sabato Sorrentino, Ulf Landmesser, Salvatore De Rosa, Daniele Torella

**Affiliations:** a Department of Medical and Surgical Sciences, Magna Grecia University, Catanzaro, Italy; b Department of Cardiology, Angiology and Intensive Care Medicine, Deutsches Herzzentrum der Charite, Berlin, Germany; c Cardiovascular Research Center, Magna Grecia University, Catanzaro, Italy; d Heart Center Lahr, Internal Medicine and Cardiology, Lahr, Germany; e Minneapolis Heart Institute and Minneapolis Heart Institute Foundation, Abbott Northwestern Hospital, Minneapolis, Minnesota, USA; f DZHK (German Centre for Cardiovascular Research), Berlin, Germany; g Berlin Institute of Health (BIH), Berlin, Germany

**Keywords:** calcified coronary lesions, intravascular ultrasound, IVUS, OCT, optical coherence tomography, percutaneous coronary intervention

## Abstract

**Background:**

The role of intravascular imaging guidance in percutaneous coronary intervention (PCI) of calcified coronary lesions remains incompletely defined.

**Objectives:**

The objective of the study was to compare intravascular ultrasound (IVUS) or optical coherence tomography (OCT) vs angiography-guided PCI in patients with calcified coronary lesions.

**Methods:**

A systematic review and frequentist network meta-analysis was conducted. Randomized and observational studies comparing IVUS-, OCT-, and angiography-guided PCI in calcified lesions were included. The primary endpoint was major adverse cardiovascular events (MACE), defined as cardiac death, target vessel myocardial infarction, and target vessel revascularization (TVR). Secondary endpoints included target vessel failure, target lesion revascularization, TVR, minimum stent area (MSA), and stent thrombosis.

**Results:**

Ten studies including 4,003 patients were included. IVUS- and OCT-guided PCI were associated with significantly lower MACE compared with angiography guidance (IVUS: relative risk [RR]: 0.56; 95% CI: 0.37-0.86; OCT: RR: 0.65; 95% CI: 0.52-0.82), with no statistically significant differences between imaging modalities. Results were consistent in randomized controlled trial–only sensitivity analyses. Stent thrombosis was also significantly lower with IVUS (RR: 0.15; 95% CI: 0.03-0.89) and OCT (RR: 0.12; 95% CI: 0.03-0.46). OCT-guided PCI was also associated with a larger MSA compared with angiography (MD +0.87 mm^2^; 95% CI: +0.03 to +1.70), whereas IVUS showed a nonsignificant trend in the same direction. No significant differences were observed for target vessel failure, target lesion revascularization, or TVR, although there was a trend in favor of imaging guidance for all outcomes. Heterogeneity was low except for MSA.

**Conclusions:**

In calcified coronary lesions, intravascular imaging is associated with lower MACE and stent thrombosis, compared with angiography guidance.

Percutaneous coronary intervention (PCI) of calcified lesions is common (18% to 24% of cases),^1^^,^^2^ and can be challenging due to poor device deliverability, impaired lesion preparation, suboptimal stent expansion, and poor short- and long-term clinical outcomes.^3^, ^4^, ^5^ Intravascular imaging has emerged as an essential tool in complex PCI. The use of intravascular ultrasound (IVUS) and optical coherence tomography (OCT) allows detailed plaque morphology assessment, including calcium distribution, thickness, and circumferential vs eccentric distribution, providing information to guide lesion preparation and stent optimization.^6^^,^^7^ Several but not all randomized and observational studies have showed that intravascular imaging guidance improves procedural outcomes, particularly stent expansion and apposition, and may translate into improved clinical outcomes compared with angiography guidance.^8^, ^9^, ^10^, ^11^ However, despite their spread across the percutaneous intervention clinical practice, the use of intravascular imaging in calcified coronary artery lesions remains limited. Therefore, the aim of this systematic review and network meta-analysis was to evaluate the impact of intravascular imaging guidance (with IVUS or OCT) on clinical and procedural outcomes in patients undergoing PCI for calcified coronary lesions.

## Methods

This systematic review and network meta-analysis was conducted in accordance to the Cochrane Collaboration and PRISMA (Preferred Reporting Items for Systematic reviews and Meta-Analyses) guidelines.^12^^,^^13^ A network meta-analysis was preferred over conventional pairwise meta-analysis to enable simultaneous comparison of IVUS-guided, OCT-guided and angiography-guided PCI, while also providing comparison between imaging modalities like IVUS and OCT, for which direct evidence remains limited.

### Study search

A comprehensive literature search was performed until February 22, 2026. Potentially eligible articles were found using PubMed/MEDLINE, Scopus and Web of Science. The full research strings adopted in the search strategy are provided in Supplemental Data 1.

### Study selection with inclusion/exclusion criteria

Two investigators (G.P. and G.T.) independently screened titles and abstracts for eligibility. Disagreements were addressed by consensus. Studies were considered eligible for inclusion if they met the following PICOS criteria: 1) Population: adult patients (≥18 years) undergoing PCI for angiographically or intravascular imaging-defined calcified coronary lesions; 2) Intervention: intervention performed was PCI guided by IVUS or OCT; 3) Comparator: angiography-guided PCI; 4) Outcomes: reporting at least 1 clinical or procedural outcome of interest; and 5) Study design: Randomized controlled trials or observational studies with comparative groups. Trials with broader coronary artery disease subsets were included only if specific data for calcified coronary lesions and intravascular imaging approaches were available for extraction. Exclusion criteria were case reports, small case series, reviews, editorials, conference abstracts without full data, and studies lacking a comparator group. The data extraction was conducted by co-authors G.P. and G.T.

### Outcomes

The primary outcome was major adverse cardiac events (MACE), defined as cardiac death, target vessel myocardial infarction (TVMI), and target vessel revascularization (TVR). Secondary outcomes included target vessel failure, TVR, minimum stent area (MSA), target lesion revascularization (TLR), and stent thrombosis (ST). Clinical outcomes were analyzed at the longest available follow-up reported by each study.

### Evaluation of study quality

The quality of the included studies was assessed by 2 co-authors (G.P. and G.T.). Disagreements were resolved through discussion and agreement. The risk of bias for randomized controlled trials was assessed using the Cochrane Risk of Bias 2.0 tool,^14^ whereas observational studies were evaluated using the ROBINS-I (Risk of Bias in Nonrandomized Studies of Interventions) tool.^15^

### Statistical analysis

Continuous variables were expressed as mean ± SD, whereas categorical variables were reported as percentages. A random-effects network meta-analysis was performed within a frequentist framework to compare angiography-guided, IVUS and OCT-guided PCI of calcified coronary lesions. For categorical outcomes, relative risk (RR) with 95% CI was assessed.^16^ For continuous outcomes, mean differences with 95% CIs were estimated. Between study heterogeneity was assessed using the I^2^ statistics. A continuity correction was applied in study with zero events in 1 treatment arm. Network geometry was evaluated by constructing network plots, where node size was proportional to the number of the patients, and edge thickness to the number of the direct comparisons. Consistency between direct and indirect evidence was assessed using the separating indirect from direct evidence (SIDE) approach based on back calculation. For each comparison with available direct evidence, network estimates were decomposed into direct and indirect components, and their agreement was assessed by calculating the ratio of ratios and corresponding z-tests. An exploratory meta-regression analysis was performed to evaluate the interaction between intense lesion preparation, defined as the adoption of cutting/scoring balloons, rotational/orbital atherectomy or intravascular lithotripsy, and MACE. For each study comparison, the covariate was obtained as the mean proportion across the two arms. A 2-sided *P* value <0.05 was considered indicative of statistically significant inconsistency. Consistency of the network was evaluated using both the design-by-treatment interaction model (global inconsistency) and node-splitting analyses (local inconsistency). Significant inconsistency was defined as a *P* value <0.05. The analyses were conducted using R software (version 4.3.2, R foundation for Statistical Computing).

### Treatment ranking

Treatments were ranked according to *P* scores, reflecting the probability that each strategy was superior to competing interventions for a given outcome. Higher *P*-scores indicated better relative performance. The analysis was registered in PROSPERO, the international prospective register of systematic reviews (CRD420261287517).

## Results

### Selected studies and baseline characteristics

**Figure 1:**
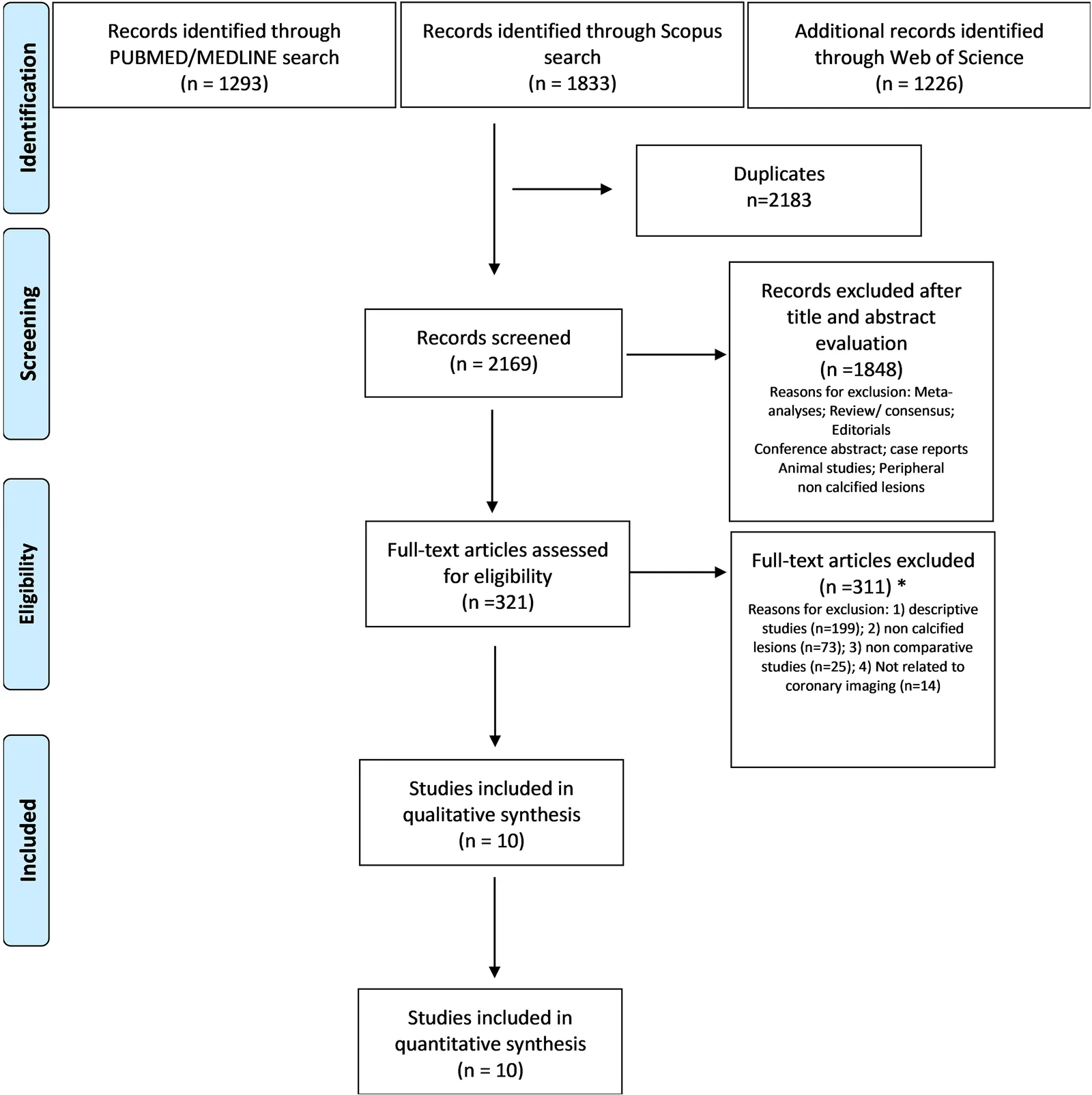
PRISMA Flowchart of Included Studies

Among the search strategy, 4,352 studies were assessed. After title and abstract screening, 321 full-text articles were evaluated. Of these, 10 studies met the inclusion criteria and were included in the network meta-analysis.^17^, ^18^, ^19^, ^20^, ^21^, ^22^, ^23^, ^24^, ^25^, ^26^ The study selection process is illustrated in the PRISMA flow diagram (Figure 1). A total of 10 studies including 4,003 patients were included. Of these, 6 were randomized controlled trials and 4 were observational studies. Median follow-up duration was 15^12^, ^13^, ^14^, ^15^, ^16^, ^17^, ^18^, ^19^, ^20^, ^21^, ^22^, ^23^, ^24^ months. Included studies compared angiography-guided, IVUS-guided and OCT-guided PCI in patients with calcified coronary lesions. Definitions of calcification were based on angiographic or intravascular-imaging features. Baseline characteristics and study details are summarized in Tables 1 and 2.

**Table 1:** Baseline Characteristics of the Included Studies

Study Name or First Author	Study Type	Year	Intracoronary Imaging Comparison	Sample Size	Inclusion Criteria	Calcified Lesion Definition	Primary Endpoint	MACE Definition	Age	Male Sex (%)	Diabetes (%)	HTN	Chronic Kidney Disease	Hypercholesterolemia	Smoking
CALIPSO	RCT	2025	OCT	134	CCS, calcified lesions, OCT crossable	Angiographic (Mintz B or C)	MSA	Cardiovascular death, myocardial infarction, or need for clinically driven reintervention on the target lesion	73 (66-78)	109 (81)	51 (38)	89 (66)	6 (5)	117 (87)	18 (13)
ULTIMATE	RCT	2021	IVUS	1,448	De novo lesion requiring DES	Angiographic	TVF	Cardiac death, TVMI, and clinically driven TVR.	65.5 ± 10.4	1,065 (73.5)	443 (30.6)	1,033 (71.3)	349 (24.1)	789 (54.5)	481 (33.2)
ILUMIEN IV	RCT	2025	OCT	1,082	Complex aniographic lesion with myocardial ischemia	Angiographic: radiopacities seen only during the cardiac cycle (moderate) or without cardiac motion generally on both sides of the arterial lumen (severe)	MSA	Cardiac death, TVMI, or ischemia-driven TVR	67.15 ± 10.05	830 (76.7)	439 (40.6)	804 (74.3)	97 (9)	721 (40.6)	174 (16.1)
Kobayashi et al^20^	ROS	2020	OCT vs IVUS	196	De novo calcified lesions treated with rotational atherectomy	OCT calcium arc >180° or lesions with angiographic calcification where an imaging catheter could not pass through	% stent expansion	*Not reported*	71.4 ± 9.61	137 (69.9)	72 (36.7)	144 (73.5)	40 (20.4)	94 (48)	10 (5.1)
Kurogi et al^21^	ROS	2022	OCT vs IVUS	145	Stable and unstable angina with moderate to severe calcification	Angiographic: moderate calcification was defined as radiopacities noted only during the cardiac cycle before contrast injection. Severe calcification was defined as radiopacities observed without cardiac motion	% stent expansion	*Not reported*	75 (65-85)	99 (68.3)	67 (46.2)	125 (86.2)	15 (10.3)	101 (69.7)	13 (9)
Maehara et al^22^ ECLIPSE OCT substudy	RCT	2026	OCT	555	Patients with chronic or acute coronary syndromes undergoing PCI with severe calcification	Severe calcification by angiography was defined as the presence of radiopacities noted without cardiac motion involving both sides of the arterial wall, with total calcium length ≥15 mm extendinginto the target lesion. Severe calcium by IVI was defined as ≥270° of calcium in at least 1 IVI cross section.	MSA	Cardiac death, TVMI, or ischemia-driven TVR	69.6 ± 8.6	419 (75.5)	217 (39.1)	478 (86.1)	60 (10.8)	497 (89.5)	57 (10.3)
OCTIVUS	RCT	2023	OCT vs IVUS	2008	Patients with obstructive CAD	Angiographic	TVF	Death from cardiac causes, TVMI, or ischemia-driven TVR	64.7 ± 10.4	1,575 (78.4)	670 (33.4)	1,286 (64)	*not reported*	1,681 (83.7)	406 (20.2)
OCTOBER	RCT	2023	OCT	1,201	Angina (stable or unstable) or NSTEMI, indication for PCI and bifurcation lesion	Angiographic: moderate defined as radiopacity in the cardiac cycle before contrast injection and severe as radiopacity without cardiac motion before contrast injection.	MACE	Death from a cardiac cause, target lesion myocardial infarction, or ischemia-driven target lesion revascularization	66.3 ± 10.2	948 (78.9)	200 (16.7)	870 (72.4)	26 (2.2)	927 (77.2)	162 (13.5)
ORBIT II	ROS	2017	IVUS	443	De novo, severely calcified lesion treated with OA	Severe calcification by angiography was defined as the presence of radiopacities noted without cardiac motion involving both sides of the arterial wall, with total calcium length ≥15 mm extendinginto the target lesion. Severe calcium by IVUS was defined as ≥270° of calcium in at least 1 IVUS cross section.	MACE	cardiac death, MI, and TVR	71.4 ± 9.9	284 (64.5)	159 (36.1)	403 (91.6)	*not reported*	404 (91.8)	73 (16.6)
Teng et al^26^	ROS	2021	OCT/IVUS	79	Moderate or severe calcified lesion treated with RA	Severe calcified lesions were defined as radiopacities seen without cardiac motionbefore contrast injection usually affecting both sides of the arterial lumen, and moderate calcified lesions as radiopacities noted only during the cardiac cycle before contrast injection.	% stent expansion	Cardiac death, myocardial infarction, TVR and stent thrombosis	69.3 ± 8.75	52 (65.8)	44 (55.7)	62 (78.5)	*not reported*	30 (38)	41 (51.9)

CAD = coronary artery disease; CCS = chronic coronary syndromes; DES = drug-eluting stents; HTN = hypertension; IVI = intravascular imaging; IVUS = intravascular ultrasound; MACE= major adverse cardiovascular events; MSA = minimum stent area; NSTEMI = non–ST-segment elevation myocardial infarction; OA = orbital atherectomy; OCT= optical coherence tomography; PCI = percutaneous coronary intervention; RA = rotational atherectomy; RCT = randomized controlled trial; TVF = target vessel failure; TVR = target vessel revascularization; TVMI = target vessel myocardial infarction.

**Table 2:** Procedural and Clinical Features of the Included Studies

Study Name	Follow-Up Duration (Months)	Stent Length (mm)	Stent Diameter (mm)	Max Diameter Balloon (mm)	MACE n (%)		MSA (Mean ± SD)		CV Death n (%)		TVMI n (%)		TLR n (%)		TVF n (%)	
CALIPSO	1	40 (28-51)	3.5 (3-3.5)	3.5 (3.5-4)	6 (8.8)	5 (8.1)	6.5 (5.5-8.1)	5 (4.1-6.1)	0 (0)	0 (0)	6 (8.8)	5 (8.1)	0	0	Not reported	Not reported
ULTIMATE	36	66.46 ± 45.27	3.07 ± 1.56	3.73 ± 0.53	33 (16.2)	18 (8.6)	Not reported	Not reported	Not reported	Not reported	0	0	45 (6.3)	27 (3.8)	33 (16.2)	18 (8.6)
ILUMIEN IV	24	41.76 ± 20.89	3.19 ± 0.42	3.56 ± 0.52	25 (4.7)	15 (2.8)	5.33 ± 1.78	5.57 ± 1.86	9 (1.7)	5 (0.9)	21 (4.0)	10 (1.9)	35 (6.8)	28 (5.5)	51 (9.7)	35 (6.8)
Kobayashi et al^20^	12	33.48 ± 16.42	2.94 ± 0.4	3.04 ± 0.46	Not reported	Not reported	OCT vs IVUS	OCT: 5.3 ± 2.1 IVUS: 5.4 ± 1.7	Not reported	Not reported	Not reported	Not reported	OCT vs IVUS	OCT: 6 (7.1) IVUS: 13 (11.6)	Not reported	Not reported
Kurogi, et al^21^	12	Not reported	3.0 (2.75-3.25)	3.0 (2.75-3.25)	Not reported	Not reported	OCT vs IVUS	OCT: 5.09 (4.38-6.01) IVUS: 4.71 (3.81-5.46)	Not reported	Not reported	Not reported	Not reported	OCT vs IVUS	OCT: 0 (0) IVUS: 4 (5.1)	Not reported	Not reported
Maehara, et al^22^ ECLIPSE OCT substudy	12	38.79 ± 21.5	Not reported	3.55 ± 0.52	96 (12.6)	39 (5.0)	OCT alone	5.71 (4.55-7.17)	33 (4.6)	7 (1.2)	38 (5.1)	19 (3.4)	32 (4.6)	14 (2.5)	96 (13.2)	39 (7)
OCTIVUS	12	47.50 ± 32.30	Not reported	Not reported	OCT vs IVUS	OCT: 42 (4.2) IVUS: 54 (5.4)	Not reported	Not reported	OCT vs IVUS	Not reported	Not reported	Not reported	OCT vs IVUS	Not reported	OCT vs IVUS	Not reported
OCTOBER	24	59 (39-78)	Not reported	4.01 ± 0.02	29 (14.9)	24 (12.2)	Not reported	Not reported	Not reported	Not reported	Not reported	Not reported	Not reported	Not reported	Not reported	Not reported
ORBIT II	36	Not reported	Not reported	Not reported	98 (24.2)	5 (14.3)	2.9 ± 0.5	2.9 ± 0.6	28 (7.0)	1 (2.9)	45 (11.2)	3 (8.6)	(8.2)	(2.9)	(3.5)	(0)
Teng et al^26^	18	62.59 ± 21.57	2.96 ± 0.34	3.26 ± 0.40	OCT vs IVUS	OCT: 1 (3.1) IVUS: 3 (6.4)	OCT vs IVUS	OCT: 5.6 ± 1.3 IVUS: 5.7 ± 1.2	OCT vs IVUS	OCT: 0 (0) IVUS: 2 (4.3)	OCT vs IVUS	OCT: 1 (3.1) IVUS: 0 (0)	Not reported	Not reported	Not reported	Not reported

Study Name	TVR, n (%)		Stent Thrombosis, n (%)		Contrast Induced Nephropaty, n (%)	
	Angiography	Imaging	Angiography	Imaging	Angiography	Imaging
CALIPSO	Not reported	Not reported	Not reported	Not reported	Not reported	Not reported
ULTIMATE	Not reported	Not reported	8 (3.9)	1 (0.5)	Not reported	Not reported
ILUMIEN IV	39 (5.1)	10 (1.8)	8 (1.5)	1 (0.2)	Not reported	Not reported
Kobayashi, et al^20^	Not reported	Not reported	Not reported	Not reported	Not reported	Not reported
Kurogi, et al^21^	Not reported	Not reported	Not reported	Not reported	Not reported	Not reported
Maehara, et al^22^ ECLIPSE OCT substudy	39 (5.1)	10 (1.8)	10 (1.3)	1 (0.2)	Not reported	Not reported
OCTIVUS	OCT vs IVUS	Not reported	OCT vs IVUS	OCT: 0 (0) IVUS: 2 (0.2)	OCT vs IVUS	OCT: 14 (1.4) IVUS: 15 (1.5)
OCTOBER	Not reported	Not reported	Not reported	Not reported	Not reported	Not reported
ORBIT II	14 (3.4)	0 (0)	Not reported	Not reported	Not reported	Not reported
Teng et al^26^	OCT vs IVUS	OCT: 0 (0) IVUS: 1 (2.1)	OCT vs IVUS	OCT: 0 (0) IVUS: 1 (2.1)	OCT vs IVUS	OCT: 1 (3.1) IVUS: 0 (0)

CV = cardiovascular; TLR = target lesion revascularization; other abbreviations as in Table 1.

### Primary outcome

**Figure 2:**
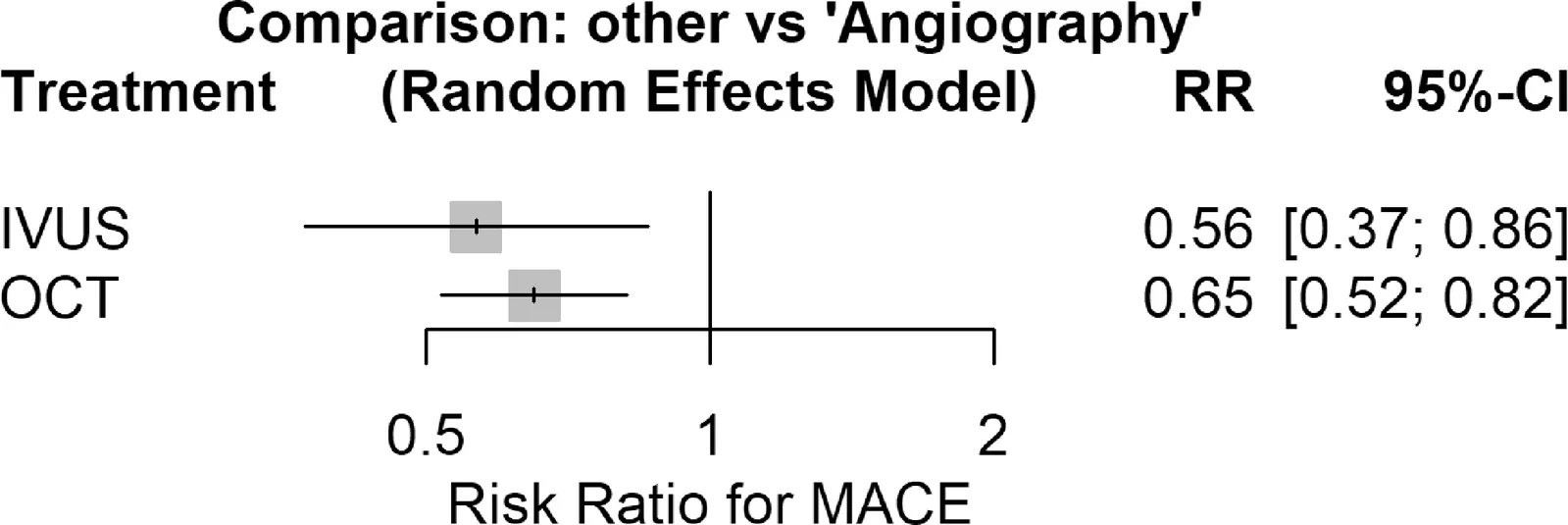
Forest Plot for the Primary Outcome of MACE IVUS = intravascular ultrasound; MACE = major adverse cardiovascular events; OCT= optical coherence tomography; RR = relative risk.

In the primary network meta-analysis, both IVUS- and OCT-guided PCI were associated with significantly lower rates of MACE compared with angiography-guided PCI (RR for IVUS: 0.56; 95% CI: 0.37-0.86; *P* = 0.007; RR for OCT: 0.65, 95% CI: 0.52-0.82; *P* < 0.001) (Figure 2). No significant differences were observed between IVUS- and OCT-guided PCI (RR: 0.89; 95% CI: 0.56-1.43). Overall heterogeneity was low (I^2^ 0%, τ^2^ between-study variance 0%), showing high consistency across included studies. For the comparison between IVUS and OCT, approximately 21% of the evidence was derived from direct head-to-head comparisons, whereas 79% came from indirect comparisons. Consistency between direct and indirect evidence with SIDE approach showed no significant inconsistency for any comparison (all *P* = 0.58), supporting agreement across the network. Treatment ranking based on *P*-scores indicated that IVUS-guided PCI had the highest probability of being the most effective strategy to reduce MACE (*P*-score 0.84) followed by OCT (*P*-score 0.65), with angiography ranking lowest (*P* score = 0.002) (Supplemental Figure 1). As the study from Teng et al also included ST in the MACE definition, it was not included in the MACE analysis, to maintain a consistent definition of the primary endpoint across included studies. An exploratory meta-regression analysis showed no significant association between the use of intense lesion preparation techniques and the treatment effect on MACE (β = −0.0015; 95% CI: –0.0097 to 0.0067; *P* = 0.71) (Supplemental Figure 2).

Sensitivity analysis restricted to randomized controlled trial (RCT) showed consistent direction and magnitude of treatment effects with the primary analysis. Both IVUS-guided PCI (RR: 0.55; 95% CI: 0.34-0.90; *P* = 0.01) and OCT-guided PCI (RR: 0.63; 95% CI: 0.49-0.81; *P* < 0.001) were significantly associated with lower rates of MACE compared with angiography-guided PCI (Supplemental Figure 3). No heterogeneity (I^2^ 0%) or inconsistency (SIDE test: all *P* = 0.79) were observed.

### Secondary outcomes

**Figure 3:**
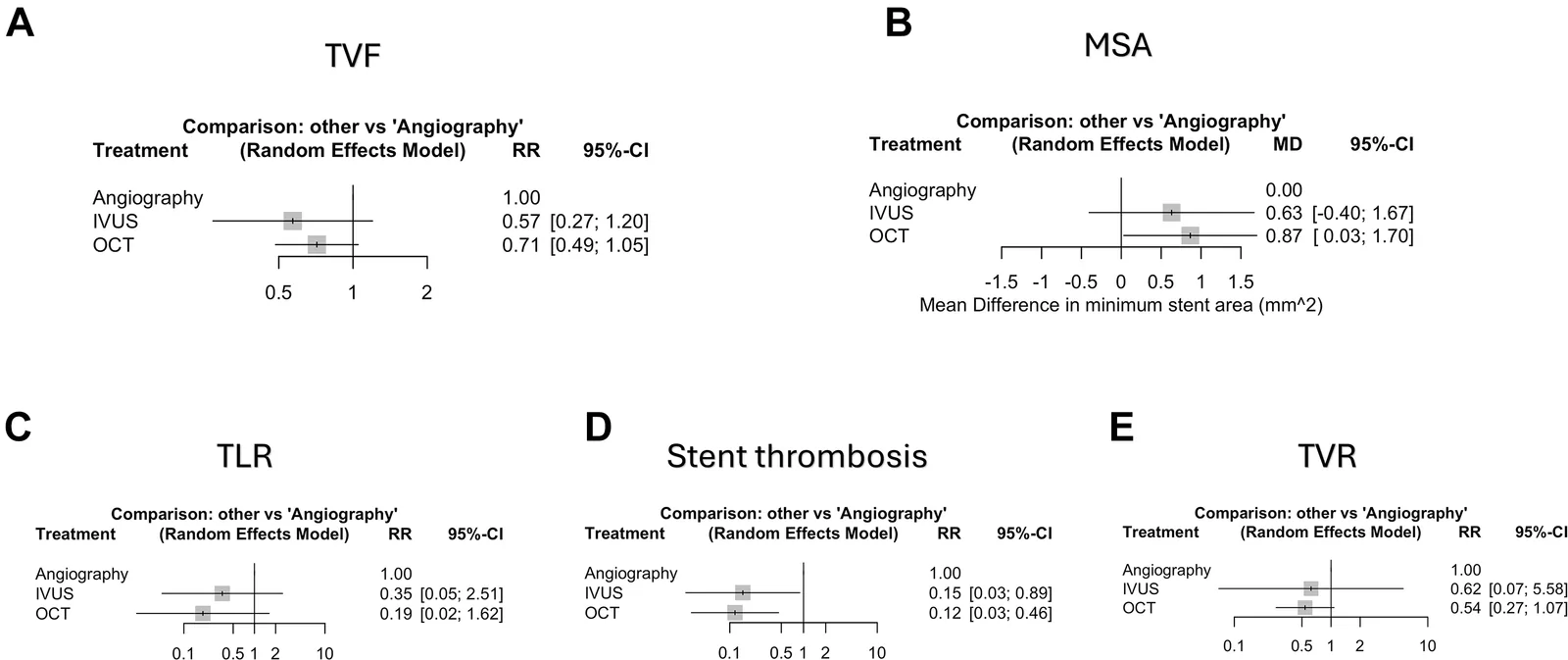
Forest Plot for Secondary Outcomes (A) TVF, (B) MSA, (C) TLR, (D) stent thrombosis, and (E) TVR. MSA = minimum stent area; TLR = target lesion revascularization; TVF = target vessel failure; TVR = target vessel revascularization; other abbreviations as in Figure 2.

IVUS use was not associated with significantly lower target vessel failure (RR: 0.57; 95% CI: 0.27-1.20; *P* = 0.14) (Figure 3A) and neither was OCT guidance (RR: 0.71; 95% CI: 0.49-1.05; *P* = 0.09). No statistically significant difference was observed between IVUS-guided and OCT-guided PCI (RR: 0.91; 95% CI: 0.41-1.99). No evidence of heterogeneity (I^2^ 0%) or inconsistency (SIDE test all *P* = 0.42) was observed. Comparison between IVUS and OCT derived at 6% from direct evidence. *P* score was 0.82 for IVUS, 0.63 for OCT, and 0.06 for angiography, respectively (Supplemental Figure 4). In MSA analysis, OCT-guided PCI was associated with a larger MSA compared with angiography guidance (MD + 0.87 mm^2^; 95% CI: +0.03 to +1.70; *P* = 0.042) (Figure 3B), whereas indirect comparison between IVUS- and angiography-guided PCI suggested a numerical trend in favor of IVUS (MD + 0.76 mm^2^: 95% CI: –0.29 to + 1.81; *P* = 0.15). No statistically significant difference in MSA was observed between IVUS-guided and OCT-guided PCI (MD −0.11 mm^2^; 95% CI: –0.73 to 0.52). Substantial heterogeneity was observed (I^2^ 82.1%). Ranking analysis showed the highest value for OCT (*P* score 0.80), followed by IVUS (*P* score 0.64), with angiography ranking last (*P* score 0.05) (Supplemental Figure 5). Comparison between IVUS and OCT was entirely provided by direct evidence. Four studies were included in the TLR analysis. Compared with angiography-guided PCI, OCT, and IVUS guidance were associated with numerically lower risk of TLR (RR for OCT: 0.19; 95% CI: 0.02-1.62; *P* = 0.12; RR for IVUS: 0.35: 95% CI: 0.05-2.51; *P* = 0.29) (Figure 3C) (*P* scores 0.92 for OCT, 0.46 for IVUS, and 0.10 for angiography) (Figure 3, Supplemental Figure 6). No statistically significant difference was observed between IVUS-guided and OCT-guided PCI (RR: 1.89; 95% CI: 0.77-4.63). Comparison between IVUS and OCT derived entirely from direct evidence. ST rates were significantly lower with both OCT (RR: 0.12; 95% CI: 0.03-0.46; *P* = 0.002) (Figure 3D) and IVUS (RR: 0.15; 95% CI: 0.03-0.89; *P* = 0.03; *P* scores 0.79 for OCT, 0.69 for IVUS, and 0.01 for angiography) (Figure 3, Supplemental Figure 7) compared with angiography. No statistically significant difference was observed between IVUS-guided and OCT-guided PCI (RR: 0.94; 95% CI: 0.07-11.86). No heterogeneity was observed (I^2^ 0%). Comparison between IVUS and OCT derived at 39% from direct evidence, with no inconsistency observed through SIDE approach (*P* = 0.70). Finally, compared with angiography-guided PCI, intravascular imaging guidance was associated with numerically lower risk of TVR (RR for OCT: 0.54; 95% CI: 0.27-1.07; *P* = 0.07; RR for IVUS: 0.62; 95% CI: 0.07-5.58; *P* = 0.67) (Figure 3E) (*P* score 0.75 for OCT, 0.55 for IVUS, and 0.18 for angiography) (Figure 3, Supplemental Figure 8). No statistically significant difference was observed between IVUS-guided and OCT-guided PCI (RR: 1.15; 95% CI: 0.13-10.48). Approximately 46% of the IVUS-versus-OCT estimate was provided by direct evidence, with no significant disagreement between direct and indirect estimates (SIDE *P* = 0.64). Sensitivity analyses for RCTs only showed consistent results with the primary analyses (Supplemental Figures 9 to 12).

### Study quality

Included studies showed a low to moderate risk of bias for the RCTs and a moderate to serious risk of bias for nonrandomized studies (Supplemental Figure 13). The observed heterogeneity was low for all the outcomes, except for MSA (I^2^ 82%). SIDE assessments did not show inconsistencies among the performed analyses (Supplemental Figure 14).

## Discussion

In this network meta-analysis of patients undergoing PCI for calcified coronary lesions, intravascular imaging guidance with IVUS and OCT was associated with significantly lower MACE and ST compared with angiography-guided PCI (Central Illustration). Moreover, both IVUS and OCT guidance resulted in a larger post-PCI MSA. Finally, imaging guidance showed favorable trends toward lower rates of target lesion and TVR, despite not reaching statistical significance. Collectively, these findings extend previous evidence from mixed-complexity population and support the incremental value of intravascular imaging in a challenging coronary artery disease subset like calcified coronary lesions. Calcified coronary lesions represent one of the most challenging scenarios in contemporary interventional practice. Calcification may impede equipment delivery and adequate lesion preparation, limit stent expansion, increase the risk of malapposition, and is associated with worse clinical outcomes.^27^^,^^28^ In this context, angiography guidance alone provides limited information regarding calcium distribution, thickness, and circumferential vs eccentric distribution, often leading to suboptimal procedural results. In our analysis, both IVUS and OCT-guided PCI were associated with a significant reduction in MACE compared with angiography. These findings are in line with prior randomized and observational studies showing improved outcomes with intravascular imaging but extend this evidence specifically in a specific high-risk population like patients with calcified coronary lesions.^29^, ^30^, ^31^, ^32^ The observed results likely derive from a more accurate lesion preparation by intravascular imaging, with appropriate calcium modification observed and adequate equipment choice, improved stent size, and deployment. Intravascular imaging allows operators to tailor plaque modification, particularly in calcified plaques, with the possibility to assess calcium thickness, distribution, eccentricity, and depth.^33^ Particularly, despite many of the included studies used advance debulking techniques in both arms, in the study from Kobayashi et al. OCT guidance allowed a more accurate device sizing, with a significantly larger and a higher number of rotational atherectomy burrs. Accordingly, in both ILUMIEN IV and CALIPSO studies a significantly higher usage of advanced debulking techniques was observed in the OCT-guided arms. This mainly results in more efficient plaque modification, with reduced incidence of underexpansion and better procedural optimization. However, we performed an exploratory meta-regression analysis which did not show a significant interaction of intense debulking techniques with the primary outcome. This analysis should be interpreted cautiously and exploratory, as the limited small number of available studies that may have reduced statistical power. However, this hypothesis-generating finding suggests that the clinical benefit derived from intravascular imaging may not be merely related to the higher usage of intense debulking, but probably reflects an improved lesion characterization, device selection, stent sizing and identification of procedural complications. Although the available evidence formed a relatively sparse network, no significant inconsistency between direct and indirect estimates was detected, supporting the validity of network assumptions. However, the comparison between IVUS and OCT should be interpreted with caution because it was based on a limited number of direct comparisons. Accordingly, the absence of statistically significant differences between IVUS and OCT should not be interpreted as evidence of therapeutic equivalence.

**Central Illustration:**
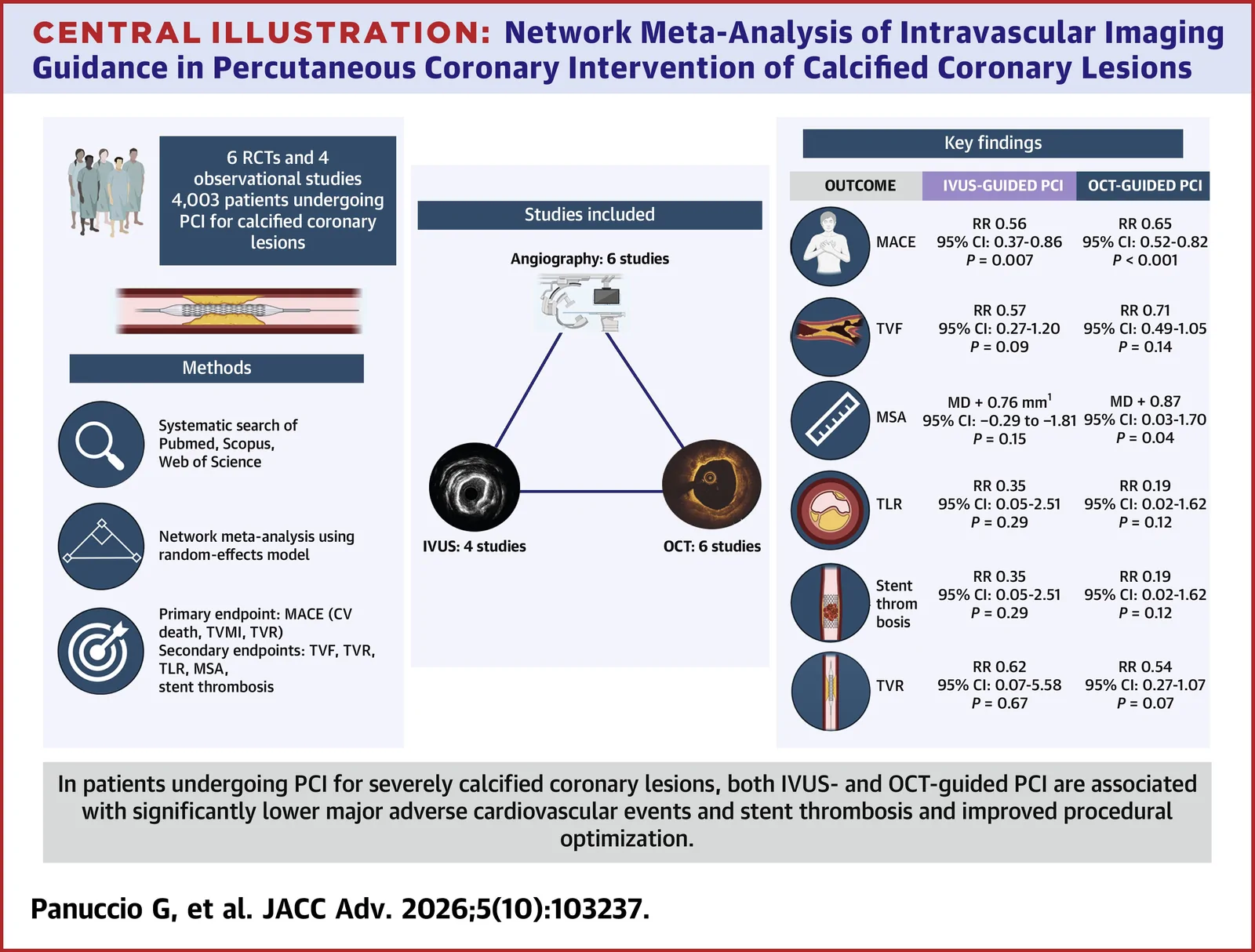
Network Meta-Analysis of Intravascular Imaging Guidance in Percutaneous Coronary Intervention of Calcified Coronary Lesions CV = cardiovascular; IVUS = intravascular ultrasound; MACE = major adverse cardiovascular events; MSA = minimum stent area; OCT= optical coherence tomography; PCI = percutaneous coronary intervention; RCT = randomized controlled trial; RR = relative risk; TLR = target lesion revascularization; TVF = target vessel failure; TVR = target vessel revascularization; TVMI = target vessel myocardial infarction.

One of the most relevant findings of this study is the significantly lower risk of ST associated with IVUS- and OCT-guided PCI. ST is a rare but serious complication after PCI and is often linked to stent deployment practice. Particularly, factors like stent underexpansion, under sizing, malapposition, and edge dissections are frequently related to higher risk of ST and are highly prevalent in calcified coronary lesions.^34^ Intravascular imaging enables systematic detection of these stent-related complications, thus reducing the risk of developing ST. Postprocedural MSA is a well-established predictor of restenosis and adverse clinical outcomes.^35^^,^^36^ In this study, OCT-guided PCI was associated with the largest MSA followed by IVUS, with angiography ranking last. OCT provides superior axial resolution and enables precise identification of calcium morphology, microfractures after plaque modification, and stent malapposition. This high-resolution imaging may facilitate more aggressive and targeted lesion preparation and postdilation, resulting in superior stent expansion. Our results suggest that the systematic use of imaging guidance in calcified lesions may substantially improve stent durability, reinforcing guideline recommendations favoring intravascular imaging in complex coronary anatomies. The relatively high heterogeneity observed for MSA is likely explained by several procedural factors. Postprocedural MSA is influenced by differences in intravascular imaging acquisition and analysis protocols, lesion preparation strategies, the use of intense debulking practices, postdilation techniques, operator experience, and optimization criteria across studies. Moreover, MSA was assessed using either IVUS or OCT, which differ in spatial resolution and measurement methodology, potentially contributing to additional between-study variability.

With respect to TVR and TLR, imaging-guided approaches showed favorable trends compared with angiography guidance. These not statistically significant findings are likely related to limited statistical power, low event rates, and small number of available studies. Nevertheless, the observed trends are directionally consistent with improved stent deployment, supporting a potential long-term benefit of imaging guidance. The reduction in MACE observed with intravascular imaging guidance was not attributable to a single individual component but rather reflected consistent favorable trends across several ischemic outcomes, including cardiac death, TVMI, and TVR. This suggests that the overall clinical benefit is likely multifactorial and related to improved lesion preparation and PCI optimization rather than to a single mechanism. The observed reduction of MACE, including its individual components, is biologically plausible. Intravascular imaging allows more accurate assessment of calcium morphology, allowing appropriate lesion preparation, and optimizing stent sizing, expansion, apposition and landing zone selection. These procedural improvements may reduce the incidence of TVMI and other ischemic complications. Moreover, intravascular imaging may also reduce thrombotic complications including ST, which was significantly lower in the intravascular imaging-guided arms as showed in our secondary endpoint analysis, and which may ultimately contribute to lower cardiac death rates. However, these mechanisms remain biologically plausible explanations of the observed associations and should not be interpreted as evidence of a direct causal relationship. Importantly, IVUS and OCT should not be considered as therapeutic intervention per se. Rather, they represent imaging-guided PCI strategies that influence clinical outcomes by enabling accurate lesion characterization and supporting operator decisions regarding lesion preparation, calcium modification, device selection, stent sizing, landing zone selection, and stent optimization. Therefore, the clinical benefit observed in this analysis is likely related to the optimization of PCI guided by intravascular imaging rather than to the imaging modality itself. As far as we know, this is the first network meta-analysis evaluating the impact of intravascular imaging guidance in the specific context of calcified coronary lesions and separately including IVUS and OCT. Recently, Carvalho et al^37^ provided a network meta-analysis on intravascular imaging in complex coronary lesions. Despite they included calcified coronary lesions as a subgroup, they only reported data regarding MACE. In contrast, the present study was specifically designed to address the particular subset of calcified coronary lesions, a setting with a greater dependence on intravascular imaging for procedural planning and optimization. Beyond evaluating MACE, our analysis specifically investigated procedural and lesion-oriented outcomes that are particularly relevant in calcified PCI, including MSA and ST. To our knowledge, this is the first network meta-analysis in this specific setting to demonstrate a lower rate of ST with intravascular imaging guidance, while simultaneously assessing procedural outcomes like MSA. By simultaneously evaluating procedural (MSA) and downstream clinical outcomes, including ST, our analysis provides mechanistic insights into how procedural optimization achieved through intravascular imaging may translate into improved long-term clinical outcomes. Furthermore, our study provides the most comprehensive synthesis of the currently available evidence in this highly selected clinical setting. Although the available evidence formed a relatively sparse network, no significant inconsistency between direct and indirect estimates was detected, supporting the validity of network assumptions. However, the comparison between IVUS and OCT should be interpreted with caution because it was based on a limited number of direct comparisons. Although angiography-guided PCI remains widely used, our data suggest that reliance on angiography in this high-risk population may expose patients to avoidable adverse events. Both IVUS and OCT should be considered integral components of contemporary PCI strategies for calcified coronary lesions, particularly in complex anatomies and high-risk clinical settings.

### Study Limitations

Some limitations should be acknowledged. First, the number of available studies for some secondary outcome was limited, resulting in reduced statistical power. Second, 4 studies were observational and not randomized. We acknowledge the inherent limitation associated to the combination of RCTs and observational studies. Although it may introduce residual confounding, this approach allowed us to capture the currently available evidence in a clinical scenario like calcified coronary lesions, which is under-represented in RCTs. However, sensitivity analyses excluding nonrandomized studies showed consistent results with our main findings. Third, for some procedural outcome like MSA the comparison of IVUS vs angiography was only indirect; this could be the explanation for the numerical trend in favor of IVUS without reaching statistical significance. Fourth, variation in lesion definition, imaging protocols, calcium modification practices, and operator expertise may have contributed to heterogeneity. Fifth, the definition of calcific lesions varied across studies, including both angiography- or imaging-based definition; moreover, some of the included studies were post hoc subgroup analyses of major RCTs or prespecified calcific subgroups. Moreover, although included studies enrolled patients with calcified coronary lesions, residual differences in lesion complexity, calcium severity, operator experience, and the use of calcium modifying devices may have influenced treatment effects. Although the absence of significant inconsistency and the results of the meta-regression support the validity of network assumption, residual violations of transitivity cannot be completely excluded. Finally, patient-level data were not available, precluding adjustment for individual and procedural variables. These limitations highlight the need for further dedicated randomized trials.

## Conclusions

In patients undergoing PCI for severely calcified coronary lesions, both OCT- and IVUS-guided PCI are associated with lower major adverse cardiovascular events and ST and improved procedural optimization. Despite the limited evidence on this high-risk population, these findings provide a strong rationale for further randomized trials to progressively integrate intravascular imaging for PCI of complex calcified coronary lesions.

## Funding support and author disclosures

This work was supported by grants from the Italian Ministry of University and Research (PNRR-National Center for Gene Therapy and Drugs based on RNA Technology No. CN00000041) and from the 10.13039/501100003196Italian Ministry of Health (POS4 ‘Cal-Hub-Ria’ No. T4-AN-09; PNRRMAD-2022-12376814). Dr Brilakis has received consulting/speaker honoraria from Abbott Vascular, American Heart Association (associate editor Circulation), Boston Scientific, Cardiovascular Innovations Foundation (Board of Directors), Cordis, Elsevier, GE Healthcare, Heartflow, IMDS, Medtronic, Recor Medical, SHockwave, SIS Medical, Teleflex, and Terumo; research support from AngioWave, Boston Scientific, and GE Healthcare; he is the owner of Hippocrates LLC and Systole LLC; and the shareholder of Cleerly Health, LifeLens Technologies, Inc, MHI Ventures, Stallion Medical, and TrueVue Inc. All other authors have reported that they have no relationships relevant to the contents of this paper to disclose.
